# LARP6 Meets Collagen mRNA: Specific Regulation of Type I Collagen Expression

**DOI:** 10.3390/ijms17030419

**Published:** 2016-03-22

**Authors:** Yujie Zhang, Branko Stefanovic

**Affiliations:** Department of Biomedical Sciences, College of Medicine, Florida State University, Tallahassee, FL 32306, USA; yujie.zhang@med.fsu.edu

**Keywords:** type I collagen, posttranscriptional regulation, collagen mRNA translation, ribonucleoprotein complexes, collagen mRNA binding protein, LARP6

## Abstract

Type I collagen is the most abundant structural protein in all vertebrates, but its constitutive rate of synthesis is low due to long half-life of the protein (60–70 days). However, several hundred fold increased production of type I collagen is often seen in reparative or reactive fibrosis. The mechanism which is responsible for this dramatic upregulation is complex, including multiple levels of regulation. However, posttranscriptional regulation evidently plays a predominant role. Posttranscriptional regulation comprises processing, transport, stabilization and translation of mRNAs and is executed by RNA binding proteins. There are about 800 RNA binding proteins, but only one, La ribonucleoprotein domain family member 6 (LARP6), is specifically involved in type I collagen regulation. In the 5′untranslated region (5’UTR) of mRNAs encoding for type I and type III collagens there is an evolutionally conserved stem-loop (SL) structure; this structure is not found in any other mRNA, including any other collagen mRNA. LARP6 binds to the 5′SL in sequence specific manner to regulate stability of collagen mRNAs and their translatability. Here, we will review current understanding of how is LARP6 involved in posttranscriptional regulation of collagen mRNAs. We will also discuss how other proteins recruited by LARP6, including nonmuscle myosin, vimentin, serine threonine kinase receptor associated protein (STRAP), 25 kD FK506 binding protein (FKBP25) and RNA helicase A (RHA), contribute to this process.

## 1. Fibrotic Diseases

Fibrotic diseases are characterized by excessive and uncontrolled synthesis of extracellular matrix proteins [1,2,3,4,5]. Type I collagen represents 80%–90% of the proteins in the fibrotic matrix and deposition of type I collagen fibrils disrupts normal tissue architecture, leading to loss of function [6]. Type III collagen is another fibrilar collagen present in the matrix, but it is a minor component and forms thin fibrils [7,8,9]. Other fibrilar collagens (type V, XI, XXIV and XXVII) are present in tracing amounts and type II collagen is not found in the fibrotic matrix. Therefore, the severity of fibrotic diseases is determined by the amount of type I collagen produced. Fibrotic diseases affect millions of people worldwide, with liver fibrosis being the most common, followed by cardiac, renal and pulmonary fibrosis. Fibrotic diseases present a major health problem, because there is no cure for fibrosis, which is chronic, progressive and often fatal. Understanding the molecular mechanism leading to excessive synthesis of type I collagen would greatly help discovery of specific antifibrotic drugs.

## 2. Type I Collagen

Type I collagen is the predominant component of the extracellular matrix and the most abundant protein in vertebrates. It comprises >90% of the organic mass of the bone and is the main constituent of tendon, skin, ligaments, cornea, arterial blood vessel walls and many interstitial connective tissues [10]. In parenchymal organs type I collagen is present in small amounts to maintain the structural integrity. In under physiological conditions type I collagen is a long-lived protein. In rats, labeling with hydroxylproline by ^18^O, followed by mass spectrometry, demonstrated that the half-life of type I collagen has a range from 45 days in muscle to 244 days in intestine [11]. Pulse-chase studies with ^3^H labeled proline showed similar turnover rate of type I collagen in various tissue, with a half-life averaging 60–70 days [12]. Therefore, to maintain the steady state levels of type I collagen the replacement or constitutive type I collagen synthesis is of low rate. However, in physiological wound healing or in non-physiological reactive fibrosis, type I collagen synthesis can be upregulated several hundred fold; this is one of the characteristics of type I collagen regulation and the process is not completely understood.

Type I collagen is the major fibrillar collagen composed of two α1(I) and one α2(I) polypeptide chains encoded by unlinked loci of collagen α1(I) and α2(I) genes. Nascent procollagen polypeptides are co-translationally inserted into the lumen of the endoplasmic reticulum (ER); during this process the polypeptides undergo excessive hydroxylations of prolines and lysines and glycosylations of hydroxyl-lysines. Procollagen polypeptides contain a central, triple helix-forming domain, with approximately 1000 amino acids having a continuous Gly-X-Y (X is frequently proline and Y is frequently hydroxyproline) repeat motif, flanked by globular N-terminal and C-terminal domains [8,13,14,15]. In the lumen of the ER two α1(I) and one α2(I) procollagen polypeptides register together with the C-terminal globular domains and fold into triple helix [16,17,18]. Requiring registration of three polypeptides, the folding of type I collagen is highly concentration dependent. After assembly, the triple helices of type I procollagen are secreted into the extracellular space, where the globular domains are cleaved off and triple helices are polymerized into fibrils [19,20,21]. The arrangement fibrils are stabilized by the formation of covalent cross-links between lysine and hydroxylysine residues, which finally contribute to the mechanical resilience of the fibrils [22,23].

This part of the biosynthetic pathway of type I collagen has been well understood through many years of research. However, much less is known about the early events in the biosynthetic pathway; the molecular interactions which commit collagen α1(I) and α2(I) mRNAs for translation and regulate the coupled production of the polypeptides. These events determine the final quantitative and qualitative outcome of the biosynthesis. Elucidation of how it is possible to convert low grade constitutive synthesis into highly active synthesis is critical for understanding of fibrotic processes. Work in recent years has suggested that this is predominantly achieved by posttranscriptional regulation; by stabilization of collagen mRNAs and by stimulating their translation. Both of these processes are regulated by binding of RNA binding protein La ribonucleoprotein domain family, member 6 (LARP6) to the conserved structure found in the mRNAs encoding type I and type III collagens. This review will address the aspects of this unique regulation.

## 3. Posttranscriptional Regulation of Expression of Type I Collagen

There are multiple independent means to regulate protein expression in eukaryotic cells, such as regulation of epigenetic modifications of chromatin structure, the rate of transcription, posttranscriptional processing (5’ capping and polyadenylation), splicing of mRNA, mRNA stability, nuclear export of mRNA, subcellular mRNA localization, the rate of translation, posttranslational modifications of a protein, and protein degradation. Our thinking of how eukaryotic cells control gene expression had been influenced for a long time by the prokaryotic paradigm and had been focused on transcription. This view has been increasingly challenged by growing number of discoveries that posttranscriptional mechanisms play a significant role. Therefore, while the steady state level of protein is determined by the amount of mRNA, which is regulated by the rate of transcription, the percentage of the transcripts that are processed and transported into the cytoplasm, it is also influenced by the rate of translation of mRNA and the stability of the mRNA. The posttranscriptional regulation of gene expression allows cells to respond to environmental stimuli more quickly than *de novo* transcription. The increase in collagen expression in wound healing and fibrosis is predominantly due to increased stability of collagen mRNAs and their regulated translation [24,25,26,27].

The range of half-lives of different mRNAs varies from minutes to hours. In terminally differentiated fibroblasts collagen mRNAs have a long half-life and exhibit differential stability under different culture conditions. In rat fibroblasts, its half-life is >16 h [28]. In NIH 3T3 fibroblasts, collagen α1(I) mRNA has a half-life of 4 h in semi-confluent cells and 9 h in confluent cells. This long half-life can be further prolonged by treatment with transforming growth factor β (TGF-β); the most potent collagen inducing cytokine [29,30,31,32,33,34]. In cardiac fibroblasts, angiotensin II, a component of renin-angiotensin-aldosterone system, prolongs the half-life of collagen mRNAs indirectly via upregulation and activation of TGF-β [35,36]. The turnover rates of collagen α1(I) mRNAs can also be regulated by cell adhesion. Reattachment of fibroblasts onto solid matrix prolongs the half-life of collagen α1(I) mRNAs from 2 h in suspended cells to 8 h in adherent cells [37].

The best evidence that the increase in half-life of collagen mRNAs is critical for development of fibrosis came from studies with hepatic stellate cells (HSCs). HSCs are the major cells responsible for high level of collagen synthesis in fibrotic liver. In normal liver HSCs maintain a quiescent state and synthesize trace amount of type I collagen. However, in the presence of profibrotic stimulators, such as TGF-β, HSCs transdifferentiate into myofibroblasts-like cells, acquiring the ability to migrate and to synthesize large amounts of type I collagen [38,39,40]. *In vitro* culturing of HSCs isolated from normal rat liver is widely accepted model to study activation of type I collagen expression and this process mimics the changes in fibrotic liver [41]. Freshly isolated HSCs are in quiescent state, but after culturing for 3 days on uncoated plastic dishes the activation process is triggered and after 7 days the transdifferentiation into myofibroblasts is completed. The 50–100-fold increase in type I collage expression seen after 7 days in culture is a combination of increased transcription and increased half-life of collagen mRNAs. However, when the transcription rate of collagen α1(I) gene was measured in quiescent HSCs and myofibroblasts by nuclear run-off assay, the transcription was increased 3-fold in myofibroblasts compared to quiescent HSCs. In contrast, the half-life of α1(I) mRNA was prolonged from 1.6 h in quiescent HSCs to more than 24 h in myofibroblasts, an increase of 16-fold [26]. Considering only 3-fold increase in the rate of transcription, the posttranscriptional regulation proved to be the predominant mechanism to account for 50–100-fold increase in the steady state level of collagen α1(I) mRNA.

## 4. LARP6 Is the Specific RNA Binding Protein of Collagen mRNAs

**Figure 1:**
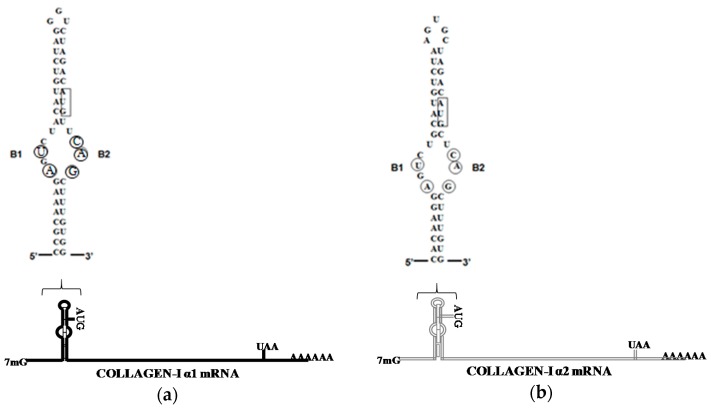
(**a**) Sequence of the 5′SL (stem-loop) of human collagen α1(I) mRNA; and (**b**) sequence of the 5′SL of human collagen α2(I) mRNA. Nucleotides critical for LARP6 binding are circled and translation start codon is boxed.

In the 5′UTR of collagen α1(I) and α2(I) mRNAs there is a 5′stem loop structure encompassing the start codon (5′ stem-loop (SL)). The 5′SL is 48 nucleotides (nt) long and is located 75–85 nt from the cap. It is well conserved in collagen α1(I) and α2(I) mRNAs of all vertebrates, implying an important function in regulating type I collagen expression. A similar sequence is not found in any other mRNA, except type III collagen mRNA. Because type III collagen is not the major determinant of the fibrotic process, the studies of regulation of this collagen by LARP6 have not been performed. Sequence of 5′SL of human collagen α1(I) and α2(I) mRNA is shown in Figure 1. The 5′SL is composed of two double stranded stems, flanking the central bulge. Cloning of LARP6 as the specific RNA binding protein which binds the 5′SL of collagen mRNAs with high affinity and specificity allowed functional characterization of this interaction. The nucleotides critical for LARP6 binding reside in the single stranded bulge, 2 nt in B1 and 3 nt in B2 region are critical for binding (Figure 1, circled). Mutation of each of these nucleotides, B1U>A, B1A>U, B2C>A, B2A>U, and B2G>U, abolished the binding of LARP6 in gel mobility shift experiments [42,43].

LARP6 contacts the 5′SL with the La module, which consists of the La motif (LaM) and the RNA recognition motif (RRM) and which is located in the N-terminus of LARP6 [42]. Although the LaM of LARP6 is similar to that of the other members of LARP super family, LARP6 utilizes some different amino acids for binding 5′SL than what the only other characterized LARP, LARP3, uses for binding RNA [44,45,46]. The most notable difference is the presence of T133 in LARP6, which is absolutely necessary for 5′SL binding [43]. Martino *et al*. used NMR to solve the structure of LaM and RRM of LARP6 and this study presents the most complete assessment of the LARP6 structure-binding relationship [47]. In these studies, LaM showed the structure of a winged-helix domain, containing an RNA binding pocket in which the contacts between the amino-acids and 5′SL RNA are established.

The RRM has a predicted β-sheet structure, but one of the loops connecting the β-strands is longer in LARP6 than in the other LARPs (loop 3, 22 amino acids compared to 3 amino acids in LARP3) [48]. This loop proved to be critical for recognition of 5′SL, because mutations of the entire loop 3 completely abolished the binding to 5′SL in gel shift experiments [43]. Simultaneous mutations of only 3 arginines within the loop 3 did not have a detrimental effect on binding [47], suggesting that the structural features other than positively charged amino-acids contribute to binding 5′SL. The structure of RRM showed that the loop 3 contains a short α helix, which appears to be firmly seated on the β-sheet face and probably precluding it from serving as the main RNA recognition platform. This suggested that loop 3 may serve as a clamp to assist the LaM in stably securing 5′SL RNA in the cleft [47]. This work revealed another important concept: for high affinity recognition of the 5′SL the correct positioning of the LaM and RRM is required, with the short linker between these domains restricting local diffusion and defining a maximum distance between the LaM and RRM domains. Replacement of the LARP6 linker with the longer linker of LARP3 had a detrimental effect on RNA binding [47]. Thus, the three piece modular construction of the La module of LARP6, representing a unique arrangement of the LaM, linker and RRM, allows for specific recognition of 5′SL. However, identification of the exact contacts between LARP6 and 5’SL RNA must await resolution of the crystal structure.

The equilibrium binding constant of LARP6 to the 5′SL of α1(Ι) mRNA and α2(I) mRNA is similar. Fluorescence polarization measurements of Kd estimated the Kd of ~0.5 nM [43], however, ITC measurements estimated the Kd of 40 nM [47]. Regardless of this discrepancy, the binding of LARP6 to 5′SL is of high affinity. Other RNA binding targets of LARP6 have not been published thus far; until such targets are identified we will consider that LARP6 is the specific RNA binding protein of collagen mRNAs. When LARP6 was pre-bound to 5′SL RNA and challenged for dissociation by adding an excess of competitor 5′SL RNA, the LARP6/α2(I)5′SL complex was more resistant to dissociation that the LARP6/α1(I)5′SL complex [43]. This suggested that binding of LARP6 to collagen α2(I) mRNA is more stable under competitive conditions that normally exist in the cell. We postulated that more stable binding to collagen α2(I) mRNA secures coupling of this mRNA into the translational pathway with collagen α1(I) mRNA for preferential synthesis of heterotrimers of type I collagen over homotrimers of α1(I) polypeptides.

Binding of LARP6 to 5′SL of collagen mRNAs has been found both in the nucleus and in the cytoplasm. Nuclear localization signal of LARP6 has been mapped to the carboxyl terminal domain, between amino acids 297 to 303 [42,49]. The localization of LARP6 in the nucleus may function as a carrier of collagen mRNAs, transporting them from the nucleus into the cytoplasm.

## 5. Role of LARP6 Binding in Fibrosis Development

The role of 5′SL in the profibrotic response was analyzed *in vitro* using HSCs in culture and *in vivo* using a knock in mouse model [50,51]. If trans-acting factors are needed for type I collagen expression in HSCs, then they can be titrated out by expressing a decoy RNA containing the binding site for these factors. Based on this premise, a molecular decoy was constructed that contained 5′SL structure at the 5′ end, followed by the sequence of U7 snRNA. The 5′SL was placed to titrate LARP6 and the U7 sequence to allow packaging with the Sm proteins to stabilize the hybrid RNA. When the decoy with 5′SL was expressed in HSCs, it reduced both collagen α1(I) and α2(I) mRNA, as well as protein expression, by 50%–60%. The control decoy lacking the 5′SL had no effect, suggesting that sequestration of LARP6 reduces type I collagen synthesis by HSCs [51].

The ultimate confirmation of the essential role of 5′SL in collagen expression was obtained when the 5′SL knock in mice were created. In these mice the collagen α1(I) gene was mutated such that nucleotides encoding for 5′SL were substituted with an unrelated sequence, while the rest of the gene was intact. The mutant gene encoded for α1(I) mRNA with the intact open reading frame (ORF) and which was expressed at normal levels; it just lacked the 5′SL regulatory element. Knock in mice were born and developed normally and type I collagen expression in the adult animals has not been drastically altered. However, when liver fibrosis was induced in homozygous mutant animals, the mice developed only 20%–30% of fibrosis seen in control mice. HSCs were also isolated from the mutant animals and subjected to activation *in vitro*. Mutant HSCs expressed low levels of type I collagen upon activation, when compared to HSCs from control mice [50]. These findings indicated that physiological amounts of type I collagen, needed for normal development and survival, can be produced when α1(I) mRNA lacks the 5′SL, but that hepatic tissues can not upregulate type I collagen after the profibrotic stimulus. The conclusion was that binding of LARP6 to 5′SL is pivotal for high level of type I collagen expression in fibrosis, while it is dispensable for constitutive synthesis, where the default synthetic pathway can provide adequate amounts of type I collagen. LARP6 knockout mice have not been created.

## 6. Stabilization of Collagen mRNAs by Interaction of LARP6 and Vimentin Filaments

Early work on posttranscriptional regulation of type I collagen expression discovered that binding of another RNA binding protein, αCP (also known as hnRNPE), to the C-rich sequence located 23 nt 3′ to the stop codon of collagen α1(I) mRNA stabilizes this mRNA [26]. Subsequently it was shown that αCP also binds C-rich regions in the 3′UTR of 15-lipoxygenase (LOX) and tyrosine hydroxylase mRNAs [52], suggesting that αCP is a common factor for stabilization of several long-lived mRNAs. In contrast to the more general role of αCP in mRNA stabilization and its preference for C-rich sequences, the sequence specific binding of LARP6 to collagen mRNAs specifically regulates the half-life of collagen α1(I) and α2(I) mRNAs.

Our work has revealed that LARP6 can interact with vimentin [53]; a protein which forms type III intermediate filaments in cells of mesenchymal origin. Collagen mRNAs can be precipitated with vimentin and association of collagen mRNAs and vimentin filaments can also be visualized by RNA FISH. RNA FISH showed that about 30% of collagen α1(I) mRNA and 50% of α2(I) mRNA are colocalized with vimentin filaments. The interaction of collagen mRNAs and vimentin filaments is 5′SL and LARP6 dependent. Mutation of the 5′SL abolished the interaction and knock down of LARP6 reduced the pull down of collagen α1(I) and α2(I) mRNAs with vimentin. This indicated that LARP6 serves as a bridge to tether collagen mRNAs to the vimentin filaments. The functional significance of this interaction was revealed when vimentin filaments were disrupted by β,β′-iminodipropionitrile (IDPN), a chemical that depolymerizes the filaments [54], or by overexpression of a dominant negative form of desmin. Desmin is related to vimentin and the dominant negative form of desmin disrupts organization of vimentin filaments [55,56,57]. In vimentin disrupted cells collagen mRNAs decayed faster, with the half-life of 5 h for collagen α1(I) mRNA and 8–9 h for α2(I) mRNAs, compared to 18 and 24 h half lives in control cells. Thus, stabilization of collagen mRNAs is achieved through interaction of LARP6 and vimentin and association of the mRNAs with the filaments.

**Figure 2:**
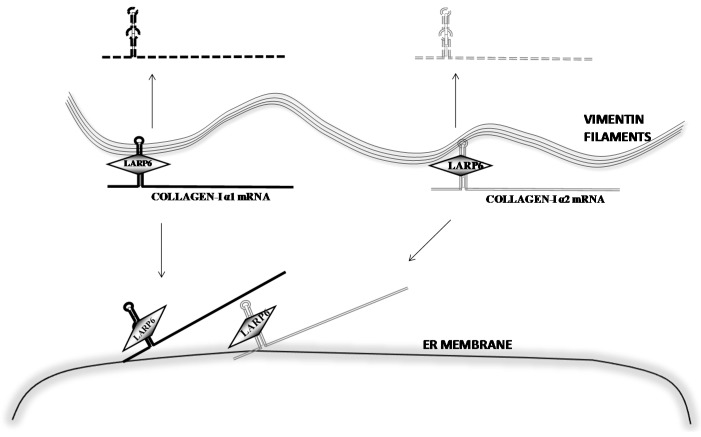
Stabilization of collagen mRNAs on vimentin filaments. Pools of collagen mRNAs on vimentin filaments can be activated for translation or subjected to degradation.

Collagen mRNAs associated with vimentin are not translated. Vimentin is not found in polysomal fractions and precipitation of vimentin does not pull down ribosomes. These results suggested that the fraction of collagen mRNAs associated with vimentin is sequestered from translation and stored as a pool of stable mRNA. Based on the estimation by RNA FISH that 30%–50% of collagen mRNAs colocalize with vimentin, we concluded that mesenchymal cells contain large stores of collagen mRNAs, which can be quickly mobilized into the translational pathway as the demand for type I collagen increases (Figure 2).

## 7. Unique Aspects of Translation of Collagen mRNAs

In general, long 5′UTRs, upstream open reading frames and secondary structure severely hamper cap-dependent ribosomal scanning and translation initiation [58,59]. mRNAs with these features are inefficiently translated, but their translational efficiency can be regulated by binding trans-acting factors, providing the ability to quickly change gene expression [60,61,62,63,64,65,66]. Collagen α1(I) and α2(I) mRNAs have two short upstream open reading frames and the secondary structure of 5′SL. Therefore, they are subjected to regulation of translation by binding of LARP6 to 5′SL. Collagen biosynthesis requires translation of two polypeptides on the membrane of the ER, but the polypeptides are not randomly translated and their production is concentrated at discrete spots [42]. A reporter protein consisting of collagen α1(I) polypeptide fused to green fluorescent protein showed accumulation at well defined foci in the ER, but the focal accumulation was observed only when the polypeptide was translated from the mRNA with 5′SL. If encoded by the mRNA without 5′SL, the accumulation of this reporter was diffuse. Knock down of LARP6 had a similar effect and converted the focal pattern of synthesis to a diffuse pattern. This indicated that both, LARP6 and 5′SL, are needed for the localized synthesis of collagen polypeptides.

**Figure 3:**
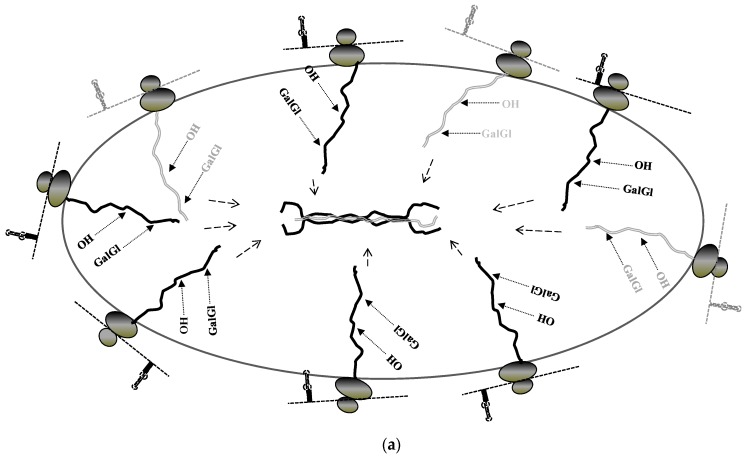

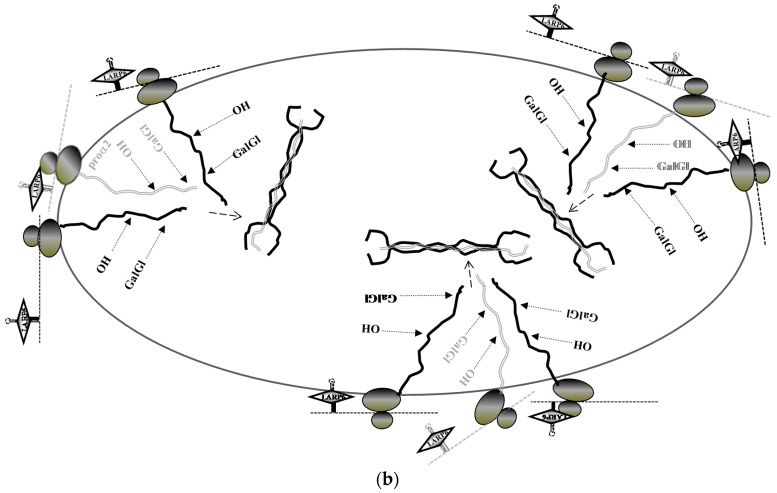
Translation of collagen mRNAs on the membrane of the ER. (**a**) LARP6 independent, constitutive synthesis of type I collagen; (**b**) LARP6 dependent, highly productive synthesis. Collagen mRNAs are shown as thin dashed lines, translating ribosomes as dark ovals, collagen polypeptides as thick lines with posttranslational modifications indicated (OH, proline and lysine hydroxylations, GalGl, glycosylations). Dashed arrows point to folding of the triple helix.

The explanation for this phenomenon came with the finding that collagen mRNAs are directly targeted to the ER membrane before the onset of translation (Figure 2). It has been well accepted that mRNA encoding secretory proteins are targeted to the ER membrane by signal recognition particle after translation of the signal peptide [67]. However, rising evidence has shown that many mRNAs are targeted to the ER membrane independently of translation and mediated by RNA binding proteins [68,69,70,71,72,73]. Collagen mRNAs showed association with the ER membrane in cells where translation initiation was inhibited by pateamine A or where polysomes were dissociated by puromycin. In contrast, mRNAs encoding two other secreted proteins, fibronectin and matrix metalloproteinase 12, showed translational dependent partitioning. Knock down of LARP6 by siRNA resulted in retention of collagen mRNAs in the cytosol [74]. These results are consistent with hypothesis that binding of LARP6 prevents premature translation of collagen mRNAs and targets the mRNAs to discrete sub regions of the ER membrane. LARP6 can interact with the ER protein translocation channel, SEC61 translocon [43], raising a possibility that collagen mRNAs are directly delivered to a subset of translocons before translation initiation. Some translocons are associated with the accessory protein TRAM2. Expression of TRAM2 is upregulated in profibrotic transformation of cells, which lead to hypothesis that TRAM2 containing translocons are the subset involved in type I collagen synthesis [75]. In this way collagen α1(I) and α2(I) mRNAs are delivered to closely positioned translocons for coordinated translation of α1(I) and α2(I) polypeptides. The coordinated synthesis is facilitated by serine threonine kinase receptor associated protein serine threonine kinase receptor associated protein (STRAP), which is recruited by interaction with LARP6 (see Section 8). The close positional synthesis of the polypeptides would assure high local concentrations for effective folding into the triple helix. It is easy to envision that this process is activated when large amounts of type I collagen are synthesized, such as in fibrosis (Figure 3).

A surprising finding that came from these studies was that the integrity of nonmuscle myosin filaments is also needed for partitioning of collagen mRNAs to the ER membrane [74]. Nonmuscle myosin forms bipolar filaments which interact with and move actin filaments. It has been extensively studied in relation to cell motility and contractility [76,77], but there have been no reports on involvement of nonmuscle myosin in translation. Depolymerization of nonmuscle myosin by the inhibitor of myosin light chain kinase, ML-7 [78], or by overexpression a dominant negative mutant of myosin light chain kinase [79] prevented partitioning of collagen mRNAs to the membrane. Inhibition of the motor function of myosin by blebistatin [80] had only minor effects, suggesting that the integrity of the filaments, rather than their motor function is required. Although it has been reported that LARP6 interacts with nonmuscle myosin [81], further studies are needed to elucidate the exact mechanism of nonmuscle myosin mediated partitioning of collagen mRNAs. HSCs highly upregulate expression of nonmuscle myosin when they transdifferentiate into myofibroblasts [82]. Similar findings were described in a mouse model of cardiac fibrosis, where fibrotic tissue was found only in the areas of the heart which showed expression of nonmuscle myosin [83]. Thus, nonmuscle myosin may not only enable motility of profibrotic cells, but also facilitate synthesis of high levels of type I collagen.

## 8. Coordinated Translation of Collagen mRNAs Is Regulated by Interaction of LARP6 and Serine Threonine Kinase Receptor Associated Protein (STRAP)

After partitioning to the ER membrane, translation of collagen mRNAs must be initiated. Serine threonine kinase receptor associated protein, STRAP, also known as upstream of N-ras interacting protein (UNRIP), was initially identified as a protein involved in regulating cap-independent translation of human rhinovirus mRNA and in assembly of snRNPs [84,85]. Homozygous deletion of STRAP generated by a gene-trap mutagenesis in mice is embryonically lethal [86]. STRAP has no kinase activity or RNA binding activity, but contains 7 tryptophan-aspartic acid (WD) domains, which are responsible for protein-protein interactions [87,88,89]. LARP6 interacts with the amino acids 294-338 in the C-terminus of STRAP, which are outside of the WD repeats [90]. For the interaction with STRAP LARP6 utilizes a conserved sequence motif in the C-terminus termed the LaM and S1 associated motif (LSM) [91]. This 20–30 amino acids motif is found in LARP6 of all species and was suggested to be involved in protein-protein interactions. In human LARP6 the LSM is required for interaction with STRAP [90]. The interaction with LARP6 recruits STRAP to collagen mRNAs. Immunoprecipitation of endogenous STRAP pulled down small amount of collagen mRNAs, but overexpressing LARP6 increased the amount of collagen mRNAs pulled down, suggesting that LARP6 is the rate limiting factor for tethering STRAP to collagen mRNAs. In gel mobility shift experiments STRAP was found in complex with LARP6 and 5′SL RNA, although STRAP alone can not bind RNA. The recruitment of STRAP is critical for coordination of translation of collagen α1(I) and α2(I) mRNAs. Embryonic fibroblasts derived from STRAP knock out mice preferentially synthesize and secrete homotrimers of α1(I) polypeptides [90], suggesting that the STRAP knock out cells can not effectively incorporate α2(I) polypeptide into type I collagen. Other collagens, including type II, do not utilize 5′SL/LARP6 dependent mechanism, because their mRNAs do not contain the 5’SL, therefore, it is unlikely that STRAP is involved in their synthesis.

Translational control executed by STRAP was revealed by comparison of polysomal loading of collagen mRNAs in control and STRAP knock out cells [90]. In control cells only about 50% of collagen α1(I) and α2(I) mRNAs were found loaded onto polysomes, while 50% was not actively engaged in translation. This indicated that translation of collagen mRNAs is normally restricted. In STRAP knock out cells similar restriction of translation was observed for collagen α1(I) mRNA, but all of collagen α2(I) mRNAs was found on polysomes. Unrestricted translation of collagen α2(I) mRNA resulted in the inability of cells to incorporate α2(I) polypeptide into the procollagen. Reexpression of STRAP in STRAP knock out cells restored incorporation of collagen α2(I) polypeptide and secretion of the heterotrimers of type I collagen. These results suggested that translation of type I collagen mRNAs must be limited to allow coordination of production of α1(I) and α2(I) polypeptides and that recruitment of STRAP is necessary for secretion of normal procollagen heterotrimer.

## 9. RNA Helicase A Increases Translational Competitiveness of Collagen mRNA

There is always surplus of mRNAs in cells compared to the amount of translational machinery. This implicates that mRNAs must compete to be translated. As mentioned earlier, type I collagen mRNAs have two short upstream open reading frames and the secondary structure of 5′SL in their 5′UTR, therefore they are not the optimal substrates for translation initiation. To compete with the other ER membrane associated mRNA for ribosomes, LARP6 recruits another accessory factor for translation, RNA helicase A (RHA) [92].

RNA helicase A, (RHA) also known as DEIH motif helicase DHX9, is a well-conserved RNA binding protein with ATPase and RNA helicase activities [93,94]. It has been shown that RHA is required to facilitate translation initiation of structured mRNAs containing the so called posttranscriptional control element (PCE). PCE consists of a stem-loop followed by GC-rich sequences and has been first found in the 5′UTR of some retroviral mRNAs and in human JunD mRNA. RHA recognizes the secondary structure of PCE, unwinds it and enhances efficiency of translations of these mRNAs [93,94,95,96,97]. 5′SL is not a PCE and RHA can not bind the 5′SL, however, RHA can interact with LARP6. Depletion of RHA by siRNA reduced expression of collagen polypeptides, which was rescued by re-expression of RHA. The reduced expression of collagen polypeptides in the absence of RHA was due to poor loading of collagen mRNAs onto polysomes. While about 50% of collagen mRNAs is normally found on polysomes, knockdown of RHA resulted in almost complete exclusion of collagen α1(I) and α2(I) mRNAs from polysomes and their accumulation in fractions containing ribosomal subunits [92]. Therefore, LARP6 enhances the translation of type I collagen mRNAs by tethering RHA. If RHA unwinds the secondary structure of 5′SL and releases LARP6 to enhance translation elongation remains to be established.

The role of RHA in promoting synthesis of type I collagen was also evidenced during the activation of HSCs. Quiescent HSCs do not expresses detectable levels of RHA, but after their activation RHA is highly upregulated and its expression parallels that of type I collagen [92].

## 10. Interaction of LARP6 and FKBP3 Increases the Half-Life of LARP6

Little is known about how LARP6 is regulated itself in collagen producing cells and in fibrosis. Preliminary studies indicate that posttranslational modifications of LARP6 by phosphorylation play an important role in activation of LARP6 (see next paragraph). LARP6 also interacts with a molecular chaperone, 25 kD FK506 binding protein (FKBP25 or FKBP3) [98]. FKBP3 is a member of FKBP superfamily, which is involved in transcriptional regulation, ribosome biogenesis, T-cell activation, and tumor suppression [99,100,101,102]. All members of the superfamily have cis-trans prolyl isomerase (PPIase) activity and can bind immunosuppressant drugs, such as FK506. PPIase activity catalyzes the isomerization of proline (cis-trans conformational change) in peptides and proteins, regulating their folding and structure [102,103].

Knock down of FKBP3 or treatment of fibroblasts with its inhibitor, FK506, reduced expression of collagen polypeptides. The level of LARP6 protein in these cells was lower than in control cells and the half-life of LARP6 was reduced from 12 h in control cells to 6 h in FKBP3 knock down cells (unpublished). This suggested that the interaction between LARP6 and FKBP3 may be needed to stabilize LARP6 and to increase its effective concentration in the cells. At present it is not known if cis-trans isomerization of a proline in LARP6 or formation of a stable complex between LARP6 and FKBP3 is responsible for this effect.

Treatment with FK506 reduced fibrosis in an *in vivo* model of hepatic fibrosis and an inefficient translation of type I collagen mRNAs in the livers of these animals was observed [98]. Although the levels of LARP6 could not be measured in the whole liver due to the low expression of the protein, it is plausible that the impaired translation of collagen mRNAs was due to the decreased LARP6 concentrations. No inflammation was seen in this model, suggesting that a novel anti-fibrotic mechanism of FK506 involves indirect targeting of LARP6. Anti-fibrotic effect of FK506 has also been observed in a mouse model of pulmonary fibrosis [104], justifying further studies of the role of FKBP3 in LARP6 metabolism and fibrosis.

## 11. Phosphorylation of LARP6 Regulates Its Activity in Fibrosis

LARP6 is a phosphoprotein and eight phosphorylation sites have been identified. Six of these phosphorylation sites reside in the C-terminal domain of LARP6 (CTER). These sites are Ser348, Ser396, Ser409, Ser421, Ser447, and Ser451. The CTER domain is not involved in collagen mRNA binding, so CTER phosphorylations do not affect the binding to collagen mRNAs, but the interactions with other proteins. LARP6 becomes highly phosphorylated in activation of HSCs, suggesting that posttranslational modifications, rather than increase in expression, are important. Recent work indicated that Ser451 is phosphorylated by Akt and that this modification activates other phosphorylation events on LARP6 [105]. Based on the importance of LARP6 dependent mechanism of type I collagen production in fibrosis, phosphorylation of LARP6 is currently an active area of research.

## 12. Conclusions

Understanding regulation of type I collagen biosynthesis has been challenging, because of its complexity and the many steps involved, including transcription of collagen genes, translation of collagen mRNAs, modification and folding of collagen polypeptides, secretion and processing of procollagen into tropocollagen, and formation of fibrilar type I collagen. Cloning of LARP6 as the specific collagen mRNAs binding protein enabled elucidation of the early steps in collagen biosynthesis; the steps that commit collagen mRNAs to translation and define the rest of the biosynthetic pathway. LARP6 binds the collagen transcripts in the nucleus and, upon export into the cytoplasm, restricts their default translation. A fraction of transcripts is stabilized and stored on vimentin filaments and the rest is targeted to the membrane of the ER. At the ER membrane LARP6 recruits STRAP to couple translation of α2(I) mRNA to that of α1(I) mRNA, enabling the exclusive synthesis of heterotrimeric type I collagen, while tethering of RHA increases the translational competitiveness. Type I collagen can be made without participation of LARP6 when the demand for the protein is not high and this suffices for the constitutive synthesis. However, in reparative fibrosis (wound healing) or in reactive fibrosis (excessive scarring of organs) the LARP6 mechanism is activated resulting into a synthesis which is better organized and more effective. Further characterization of this mechanism will contribute to a better understanding of the physiology of wound healing and pathogenesis of fibrosis. The findings described here suggest that a hope of finding a cure for fibrosis lies in elucidation of the LARP6 dependent mechanisms of excessive accumulation of type I collagen.

## Abbreviations

LARP6: La ribonucleoproteins domain family, member 6
ER: endoplasmic reticulum
HSC: hepatic stellate cell
TGF-β: transforming growth factor β
RRM: RNA recognition motif
nt: nucleotides
LOX: 15-lipoxygenase
ORF: open reading frame
UNRIP: upstream of N-ras interacting protein
STRAP: serine threonine kinase receptor associated protein
RHA: RNA helicase A
PCE: posttranscriptional control element
FKBP25/FKBP3: 25 kD FK506 binding protein
PPIase: cis-trans prolyl isomerase
CTER: C-terminal of domain
